# Heparin-induced thrombocytopenia in a non-heparin-naive patient: a case report

**DOI:** 10.1186/s40064-015-1174-5

**Published:** 2015-08-14

**Authors:** Marion Wiegele, Dieter Adelmann, Johannes Gratz, Eva Schaden

**Affiliations:** Clinical Division of Anaesthesiology and General Intensive Care Medicine, Department of Anaesthesia, General Intensive Care and Pain Control, Medical University of Vienna, Waehringer Guertel 18-20, 1090 Vienna, Austria

**Keywords:** Heparin induced thrombocytopenia, HIT II, Argatroban, Low molecular heparin, LMWH

## Abstract

**Introduction:**

Administration of low molecular weight heparin (LMWH) is recommended for prophylaxis of venous thromboembolism in patients undergoing hip surgery. In this context, heparin-induced thrombocytopenia (HIT) type II is a complication of rare incidence but sometimes fatal outcome.

**Case description:**

A 52-year old obese patient undergoing antithrombotic therapy with Enoxaparin after hip surgery presented with a painful, swollen leg and thrombocytopenia on day eight after surgery. Medical history showed previous administration of Enoxaparin without complications 2 years ago. Further diagnostic investigation supplied evidence of multiple thromboembolic events and concomitant compartment syndrome. Administration of Enoxaparin was stopped immediately and treatment with Argatroban was initiated. Diagnosis of HIT was confirmed according to current guidelines. Despite interventional thrombectomy and fasciotomy, amputation of both lower limbs had to be performed due to ongoing necroses. After a 30-days-stay at the intensive care unit because of sepsis, respiratory and renal failure, clinical condition improved and the patient could be transferred for rehabilitation.

**Discussion and evaluation:**

HIT II is known as complication of administration of LMWH in the perioperative setting. Diagnosis results from clinical findings and platelet count. Argatroban is recommended as an alternative therapeutic anticoagulant in HIT II. Inflammation and surgical trauma are discussed as priming factors to increase risk of HIT II.

**Conclusions:**

Administration of LMWH may result in HIT II despite prior uneventful drug exposure. Except for immediate diagnosis, only consequent anticoagulation can stop the course of disease. Hence, interdisciplinary awareness is inevitable for early diagnosis and accurate therapy to prevent from a catastrophic clinical course.

## Introduction

Administration of low molecular weight heparin (LMWH) is recommended for prophylaxis of venous thromboembolism (VTE) in patients undergoing hip surgery (Falck-Ytter et al. 2012). In this context, heparin-induced thrombocytopenia (HIT) type II is a well-known, immune-mediated complication of rare incidence (1–6.5 % in orthopaedic patients, depending on type of heparin), but sometimes fatal outcome (Linkins et al. 2012; Girolami and Girolami 2006; Picker and Gathof 2004).

Risk factors—among others—include type of patient population. Especially cardiac and orthopaedic surgery is related to a higher incidence of HIT II (Dasararaju et al. 2013; Linkins et al. 2012). Warkentin et al. (2000) concluded: HIT-IgG antibodies are more likely to form in patients who undergo cardiac surgery than in orthopaedic patients, but among patients in whom antibodies do form, orthopaedic patients are more likely to develop HIT. Inflammation and surgical trauma are discussed as priming factors to increase the risk of HIT II (Krauel et al. 2012; Lubenow et al. 2010).

Diagnosis of HIT results from clinical findings and platelet count (Dasararaju et al. 2013; Linkins et al. 2012). Once HIT II is suspected, administration of LMWH has to be stopped without delay and an alternative therapeutic anticoagulant has to be initiated (Linkins et al. 2012). Progressive thrombocytopenia results from intravascular platelet activation. The magnitude of thrombocytopenia correlates with thrombotic risk (Greinacher et al. 2010). An increase in platelet count on the other hand represents effective therapeutic anticoagulation as it reflects the breakdown of platelets’ consumption within the thrombus. In contrast, substitution of platelet concentrates and heparin-like substances—which are notably included in most coagulation factor concentrates as well—can worsen the prognosis.

The absence of pathological findings within the patient`s medical history does not ensure that re-administration of LMWH may not result in HIT II. However, patients with heparin exposure within the last 100 days show an early onset (<5 days) of thrombocytopenia whereas if LMWH have been administered more than 3 months ago, the onset of thrombocytopenia seems to be indistinguishable from patients having had their initial exposure to heparin (typically day 5–10) (Lubenow et al. 2002; Dasararaju et al. 2013).

This article aims to emphasize, that except for immediate diagnosis, only consequent therapeutic anticoagulation can stop the course of disease. Hence, interdisciplinary awareness is inevitable for early diagnosis and accurate therapy to prevent from a catastrophic clinical course.

## Case description

A male 52-year-old patient (body mass index 43 U) undergoing antithrombotic therapy with Enoxaparin (Lovenox^®^) after hip surgery presented with ongoing pain, a swollen leg and isolated necrotic spots on day eight after surgery. Medical history revealed previous uneventful administration of Enoxaparin 2 years ago. Despite from normal platelet count (286G/l) at the beginning of LMWH therapy, standard laboratory parameters now showed a new onset of thrombocytopenia (33 G/l). Clinical state, results of ultrasound of the lower limb and a CT-angiography supplied evidence of a major thromboembolic event within the right arteria femoralis superficialis and concomitant compartment syndrome. Administration of Enoxaparin was stopped immediately and treatment with Argatroban (Argatra^®^) was initiated. According to the 4T’s score (8 points) and the detection of PF4-heparin antibodies via an ELISA immunoassay, HIT diagnosis was likely and finally confirmed with heparin-induced platelet aggregation assay (HIPAA).

**Fig. 1:**
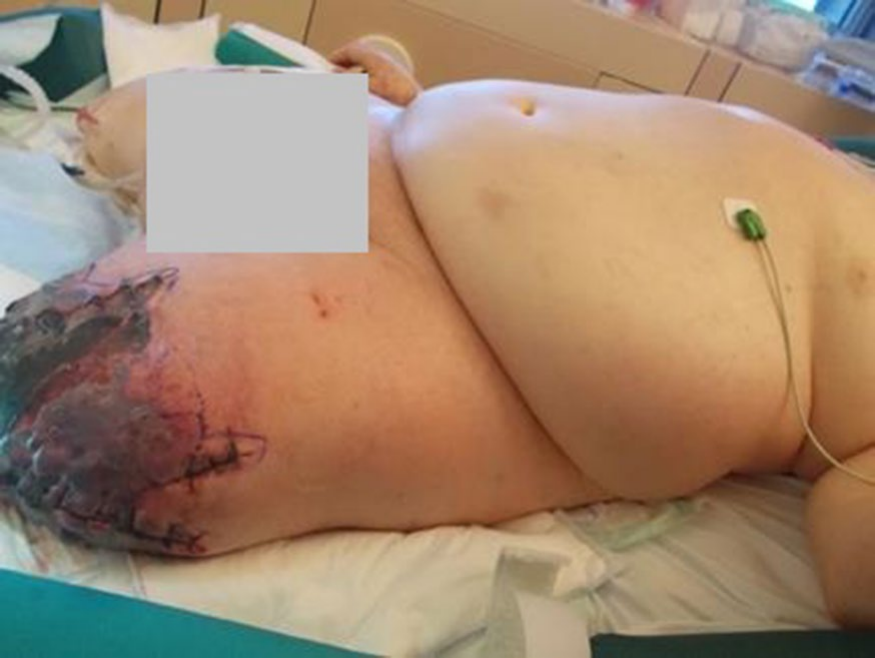
Status post recent amputation of the right leg; ongoing necrosis of the left leg despite amputation 5 days before.

**Fig. 2:**
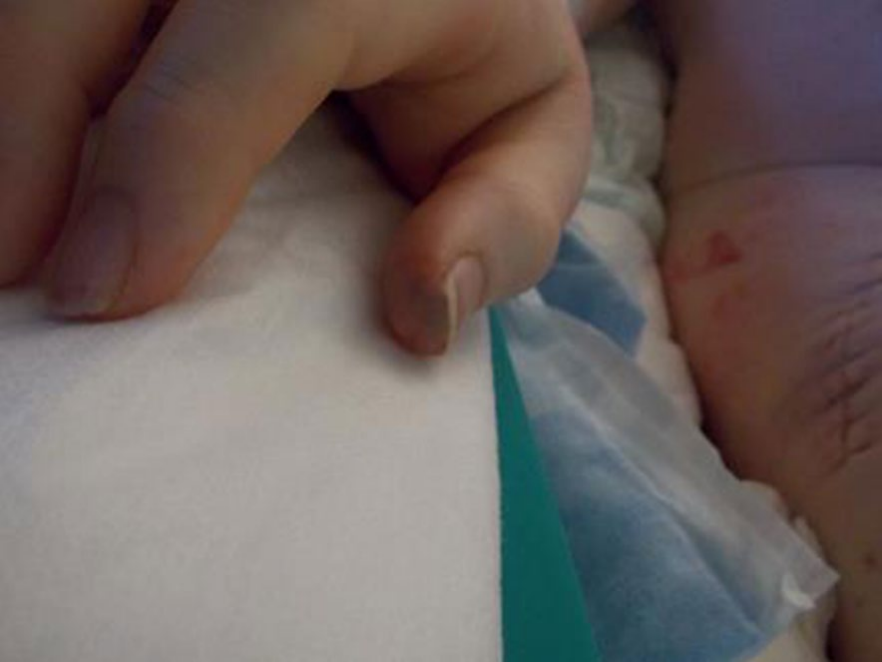
Acral necrotic spots.

Despite interventional thrombectomy and fasciotomy, amputation of the left leg had to be performed 2 days later. On day 15 after hip surgery, thrombosis of the right vena poplitea and vena femoralis superficialis required interventional thrombectomy and fasciotomy. However, this did not prevent from amputation because of ongoing necrosis of the right lower limb (Fig. 1). Acral necrotic spots indicated microembolism (Fig. 2). Due to septic shock the patient developed acute kidney failure and citrate-dialysis had to be performed. Furthermore, it took dilatative tracheotomy in order to facilitate respiratory weaning.

**Fig. 3:**
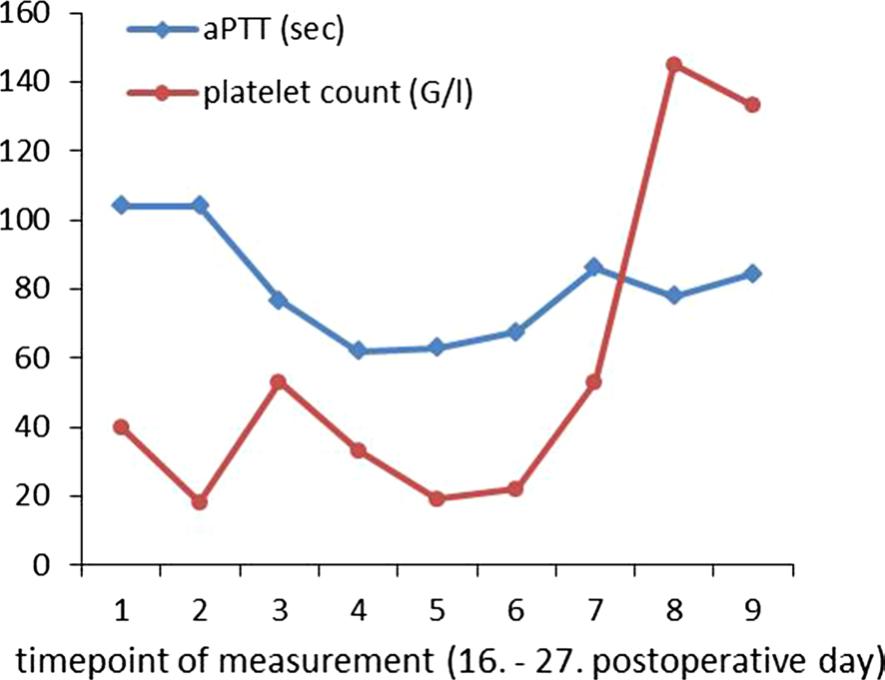
Postoperative platelet count plotted against aPTT levels.

Throughout a 30-days stay at the intensive care unit, hemodynamic and respiratory parameters stabilised and the patient regained renal function. Moreover, platelet count normalized (Fig. 3). Due to essential repetitive surgical interventions, administration of Argatroban had to be continued for at least 38 days because of—compared to vitamin k inhibitor—better perioperative controllability. Therapy with Argatroban was switched to Phenprocoumon (Marcoumar^®^) afterwards according to current bridging guidelines. The patient could be transferred to the normal ward and further rehabilitation.

## Discussion and evaluation

Although drug exposure was uneventful 2 years ago, administration of LMWH [Enoxaparin (Lovenox^®^)] resulted in a catastrophic clinical course of HIT II. Despite immediate diagnosis and adjustment to recommended therapeutic anticoagulant regime, the progress of the disease led to amputation of both lower limbs. Potential causes of this catastrophic outcome are discussed below.

The immunological pathomechanism of HIT II is complex and not yet fully elucidated. Immunoglobuline G (IgG) antibodies against platelet factor 4 (PF4)/heparin complexes play a major role in development of HIT II. PF4 on the one hand has pro- and anticoagulant function in haemostasis. Antibodies against PF4/heparin complexes generate an activation of platelets with release of procoagulant microparticles and increased generation of thrombin leading to thrombocytopenia based on platelet consumption within thrombotic formations and life-threatening arterial and venous thromboembolic events (Dasararaju et al. 2013; Linkins et al. 2012; Greinacher et al. 2010).

On the other hand PF4 is also involved in inflammatory processes due to its immune-modulating effects (Pilatova et al. 2013). Therefore, gram negative bacterial infection and trauma—which are associated with an up-regulated overall immune system—have been discussed as priming factors to increase the risk of HIT II (Krauel et al. 2012).

There is evidence, that in obese patients lipotoxicity and cytokine dysregulation create a state of low-grade systemic inflammatory response (SIRS) comparable to gram negative sepsis (Cave et al. 2008). Adipocytes trigger inflammatory pathways that lead to chronic increased levels of cytokines (e.g. TNFα, CCL 2, IL-6, IL-1β). Toll-like-receptors (TLRs) of the innate immune system are activated as well (Gregor and Hotamisligil 2011). In the reported case an obese patient was exposed to major surgical trauma. Chronic inflammatory state and trauma as a second hit possibly contributed to the onset of HIT II. To the best of our knowledge, data from randomised, controlled trials confirming this hypothesis are missing so far.

According to the patient`s medical history he underwent administration of Enoxaparin 2 years earlier in the context of surgical intervention of his shoulder. It remains speculative, if antibodies of a previous subclinical HIT could have caused the recurrence of the disease and if routinely performed monitoring of platelet count would have allowed diagnosis prior to clinical manifestation.

Reviewing current literature there is no evidence of remaining HIT antibodies 2 years after the administration of LMWH. Moreover, patients who are re-exposed to LMWH usually develop “early onset thrombocytopenia” (<5 days) due to prior immune response (Linkins et al. 2012; Lubenow et al. 2002). This was not the case in our patient who presented with new onset of thrombocytopenia on day eight after surgery. Referring to data of Lubenov and colleagues, patients with a previous exposure to heparin more than 3 months earlier had a time pattern of onset of thrombocytopenia indistinguishable from patients having had their initial exposure to heparin (Lubenow et al. 2002). This fact can be explained by the unique transient character of IgG antibodies in HIT II with a medium time to disappearance of 50–80 days (Lubenow et al. 2002). Upon administration of LMWH monitoring of platelet count should be performed daily starting on day one in patients who underwent prior treatment with heparin or LMWH within 3 months. In all other patients measuring platelet count earliest on day five is sufficient (Linkins et al. 2012). Furthermore, there is evidence of thrombocytopenia in patients undergoing hip surgery within the first two postoperative days due to reasons not related to HIT (Lubenow et al. 2002).

In the reported case clinical and laboratory findings led to immediate initiation of evidence based therapy. Following the current ACCP guidelines Argatroban (Argatra^®^)—a hepatobiliary eliminated direct thrombin inhibitor—is recommended as an alternative anticoagulant for therapy of HIT II (grade 1C). (Linkins et al. 2012) Argatroban dosing should be monitored with activated partial thromboplastin time (aPTT). According to the manufacturer’s guide one should go for a 1.5–3 fold increase of the aPTT basic level. Due to transient impaired liver function, septic status and repetitive surgical interventions during the first days, aiming for recommended aPTT levels was contraindicated with regard to increased bleeding risk in our patient. There is evidence of safely performed reduction of dose rates in critically ill patients (Saugel et al. 2010; Link et al. 2009). In addition to closely performed monitoring of aPTT levels we quantified clearance of indocyanine green (ICG) to assess hepatic function as mentioned by Link et al. (2009). ICG-PDR (ICG-plasma disappearance rate) levels were PDR 4.8 %/min and R15 48.7 %. However, we could not achieve significant increase in platelet count until aPTT levels reached the upper bound of the recommended levels (Fig. 3). Considering that the magnitude of thrombocytopenia correlates with thrombotic risk it seems, that an increase in platelet count represents effective dosage of Argatroban reflecting the breakdown of the vicious circle of consumption of IgG-coated platelets within thrombotic formations. (Greinacher et al. 2010).

## Conclusion

Administration of LMWH in order to prevent from thromboembolic events can result in HIT II despite prior uneventful drug administration.

Except for immediate diagnosis, only consequent anticoagulation can stop the course of the disease. Hence, interdisciplinary awareness is inevitable for early diagnosis and accurate therapy to prevent from a catastrophic clinical course.

## Abbreviations

ACCP: American College of Chest Physicians
aPTT: activated partial thromboplastin time
ELISA: enzyme linked immunosorbent assay
HIPAA: heparin-induced platelet aggregation assay
HIT: heparin-induced thrombocytopenia
ICG: indocyanine green
ICG-PDR: ICG-plasma disappearance rate
IgG: Immunoglobuline G
LMWH: low molecular weight heparin
PF4: platelet factor 4
SIRS: systemic inflammatory response
TLRs: toll-like receptors
VTE: venous thromboembolism
